# Fetal and neonatal brain protection at term—The role of translational experimental models

**DOI:** 10.1111/aogs.70098

**Published:** 2025-11-20

**Authors:** Juulia Lantto, Panu Kiviranta, Juha Voipio

**Affiliations:** ^1^ Division of Obstetrics & Gynecology, Department of Clinical Science, Intervention and Technology (CLINTEC) Karolinska Institutet Stockholm Sweden; ^2^ The Finnish Medical Society Duodecim Helsinki Finland; ^3^ Kuopio Pediatric Research Unit University of Eastern Finland & Kuopio University Hospital Kuopio Finland; ^4^ Faculty of Biological and Environmental Sciences, Molecular and Integrative Biosciences Research Program University of Helsinki Helsinki Finland

Severe birth asphyxia (BA) and the resulting hypoxic–ischemic encephalopathy (HIE) remain one of the main causes of neonatal brain injury and mortality in term infants globally.^1^ Hypoxic–ischemic brain injury progresses gradually, providing windows of opportunity for the development of more effective therapeutic interventions.^2^, ^3^ Translational experimental research on BA‐HIE is essential for identifying novel treatments, ideally focusing on interventions applicable in low ‐and middle‐income countries as well, where BA is most prevalent.^1^ Therefore, experimental models of BA need to be well characterized to understand the systemic and cerebral physiological interactions leading to HIE.

The pathophysiology of HIE develops in phases.^3^ Depending on its severity, the hypoxic–ischemic insult causes primary neuronal death by thoroughly studied cellular mechanisms. Birth and resuscitation are followed by the latent phase of up to 6–15 h that is characterized by recovery of oxidative energy metabolism, continued excitotoxicity, acute inflammation, and brain hypoperfusion. It provides an optimal window for therapeutic interventions, as the secondary mechanisms of injury are either already progressing or triggered during this phase. Seizures are one of the hallmarks of the secondary phase that lasts up to days and during which multiple pathogenic mechanisms progress. The tertiary phase extends from weeks to years and reveals the motor, cognitive, and behavioral consequences of the insult.

Despite extensive research into understanding the pathophysiological mechanisms of BA‐HIE‐induced brain injury and the search for novel neuroprotective interventions, the treatment methods currently in use remain inadequate. For term neonates, therapeutic hypothermia (TH) remains the only established intervention for moderate to severe HIE, available mainly in specialized centers.^3^ Additionally, TH is not globally and equitably accessible, and even where available, term brain injury may result from multiple causes — including acute, subacute, or chronic hypoxia, infection, inflammation, genetic or metabolic disorders, intracranial hemorrhage, and stroke. Therefore, this complexity underscores the need for investigating new approaches to protect the fetal and neonatal brain at term.

Much of basic and translational research has been conducted using animal models. However, choosing an appropriate model poses several challenges, including species differences, alignment of neurodevelopmental stage with human neonates, and reliable induction of asphyxia insults that replicate intrapartum conditions.^4^ No experimental model perfectly replicates human BA‐HIE, which underscores the value of complementary animal models.

Rats and mice are altricial species with brain development corresponding to preterm and full‐term human neonates at postnatal days P5–6 and P10–11, respectively.^2^ On the other hand, the most widely used large animal model species (sheep and piglets) are precocial, born with relatively mature brains.^2^ Such species differences must be considered in experimental work and when translating results to humans.

Rodent models provide data more easily and inexpensively compared to large animal models. Commonly used rodent models of BA‐HIE involve either systemic hypoxia alone or apply it following unilateral carotid artery ligation, without restricting carbon dioxide clearance. A more recent rodent model simulates BA by lowering oxygen and elevating carbon dioxide in inhaled gas to induce systemic hypoxia and hypercapnia,^5^ thereby replicating the impaired gas exchange. This difference between models is noteworthy because every vaginal birth involves some degree of hypoxia and hypercapnia together, which trigger systems‐level endogenous protective mechanisms often referred to as the peripheral chemoreflex.^2^, ^6^, ^7^, ^8^

The term fetal sheep is considered one of the most translationally relevant models for investigating fetal HIE and neuroprotection. Fetal sheep circulation and cardiovascular responses resemble those of human fetuses at term.^6^, ^7^ Furthermore, the relatively large size of the sheep fetus facilitates surgical instrumentation, and longitudinal monitoring is possible without significant risk of preterm delivery. Additionally, the effects of novel treatments can be studied during postnatal care under conditions similar to neonatal intensive care units. The fetal sheep model has played a pivotal role in establishing magnesium sulphate as a clinically validated neuroprotective treatment in preterm fetuses.^9^

At term, two thirds of the HIE cases are associated with fetal asphyxia and subacutely developing hypoxemia during active labor when blood flow to the placenta and fetus is reduced during contractions. The instrumented fetal sheep umbilical cord occlusion model replicates such consequences of uterine contractions and subacutely developing fetal hypoxemia. It is usually applied at 0.6 to 0.85 gestation to align with the neurodevelopmental stage of preterm to term human fetuses.^4^ The insult can be designed to mimic complications during human birth, but in this version of the model, the resulting pathophysiological changes must be let to progress in utero, as sheep lungs mature very late in gestation. Nevertheless, the model has been a valuable tool to bridge the gap between rodent models and human clinical trials.

Because the efficacy of TH in mitigating HIE and its lifelong consequences in severely asphyxiated newborns is relatively limited,^3^ there is a clear need for complementary treatments. Moreover, the timing of novel treatments may also be an important issue, as brain processes leading to HIE are triggered much earlier than the onset of TH. Numerous interventions have been proposed and studied mainly in animal models, including remote ischemic postconditioning, melatonin, cannabinoids, erythropoietin, caffeine etc. However, no significant benefits in humans have been demonstrated so far. Enhancing the systems‐level (as well as brain‐specific) endogenous protective mechanisms has been proposed as a more promising approach compared to a search for a single molecular target.^3^

The selection of translational animal models for HIE and neuroprotection studies must account for developmental stage, type of insult, and physiological context, as these factors critically affect the translational validity of the findings. Equally important is the perinatal transition: injury induced in utero differs from injury after the onset of postnatal circulation, and recovery before birth is not equivalent to recovery through delivery and stabilization. Optimal models must define the insult relative to transition and manage hemodynamics, ventilation, and reoxygenation in clinically relevant ways. Carbon dioxide (CO_2_) physiology deserves attention during transition. CO_2_ strongly modulates cerebral blood flow: hypocapnia constricts cerebral vessels and may precipitate hypoperfusion, whereas hypercapnia dilates them. Early, vigorous ventilation can cause sudden hypocapnia at the moment of greatest vulnerability. Avoiding iatrogenic hypocapnia and tolerating permissive hypercapnia have been proposed, consistent with the role of peripheral chemoreflex in cardiorespiratory adaptation.^10^ Experimental models that regulate CO_2_ levels may help identify strategies to optimize cerebral perfusion and support neuroprotective potential during postnatal care.

The primary clinical goal during labor is to prevent asphyxia of such severity that it causes brain injury. However, there are still no optimal or unequivocal strategies to achieve this. TH remains the standard of care for BA‐HIE in term neonates, as no superior treatment options are currently available. Therefore, many of the putative adjunctive therapies now being explored deserve systematic testing in large animal models to clarify their potential as well as possible adverse effects. Thereafter, clinical trials should also be designed around narrowly defined, homogenous patient cohorts to improve the detection of the effects of the new treatments.

Events at birth can shape outcomes across a lifetime, making even small improvements in early care highly consequential. Credible, well‐characterized experimental models integrating experimental physiology, obstetrics, neonatology, neuroscience, and biomedical engineering are needed to identify promising neuroprotective strategies. This pathway may pave the way toward more comprehensive neuroprotection.

## CONFLICT OF INTEREST STATEMENT

None declared.

## Data Availability

Data sharing not applicable to this article as no datasets were generated or analysed during the current study.
